# Supplementary material for: Connecting concepts in the brain by mapping cortical representations of semantic relations
Source: Nat Commun. 2020 Apr 20;11:1877. doi: 10.1038/s41467-020-15804-w (PMC7171176; doi:10.1038/s41467-020-15804-w)
Supplement: Supplementary file 3 — Reporting Summary [file 41467_2020_15804_MOESM3_ESM.pdf]

nature research

Corresponding author(s): Zhongming Liu
Last updated by author(s): Mar 20, 2020

Reporting Summary

Nature Research wishes to improve the reproducibility of the work that we publish. This form provides structure for consistency and transparency in reporting. For further information on Nature Research policies, seeAuthors & Referees and theEditorial Policy Checklist .

Statistics

For all statistical analyses, confirm that the following items are present in the figure legend, table legend, main text, or Methods section.

n/a	Confirmed	
☐	☒	The exact sample size (*n*) for each experimental group/condition, given as a discrete number and unit of measurement
☐	☒	A statement on whether measurements were taken from distinct samples or whether the same sample was measured repeatedly
☐	☒	The statistical test(s) used AND whether they are one- or two-sided<br>*Only common tests should be described solely by name; describe more complex techniques in the Methods section.*
☒	☐	A description of all covariates tested
☐	☒	A description of any assumptions or corrections, such as tests of normality and adjustment for multiple comparisons
☐	☒	A full description of the statistical parameters including central tendency (e.g. means) or other basic estimates (e.g. regression coefficient) AND variation (e.g. standard deviation) or associated estimates of uncertainty (e.g. confidence intervals)
☐	☒	For null hypothesis testing, the test statistic (e.g. *F*, *t*, *r*) with confidence intervals, effect sizes, degrees of freedom and *P* value noted<br>*Give P values as exact values whenever suitable.*
☒	☐	For Bayesian analysis, information on the choice of priors and Markov chain Monte Carlo settings
☐	☒	For hierarchical and complex designs, identification of the appropriate level for tests and full reporting of outcomes
☐	☒	Estimates of effect sizes (e.g. Cohen's *d*, Pearson's *r*), indicating how they were calculated

Our web collection on statistics for biologists contains articles on many of the points above.

Software and code

Policy information about availability of computer code

Data collection	Siemens Magnetom Prisma Console VE11C.
Data analysis	Matlab (2015b), Python 3.6, AFNI (17.0.01), FMRIB Software Library (6.0), FreeSurfer pipeline (5.3.0), Connectome Workbench (1.2.3), HCP minimal preprocessing pipelines (3.4.0), Gensim (3.4.0), Google's word2vec (open-source code)

For manuscripts utilizing custom algorithms or software that are central to the research but not yet described in published literature, software must be made available to editors/reviewers. We strongly encourage code deposition in a community repository (e.g. GitHub). See the Nature Research guidelines for submitting code & software for further information.

Data

Policy information about availability of data

All manuscripts must include a data availability statement. This statement should provide the following information, where applicable:

- Accession codes, unique identifiers, or web links for publicly available datasets
- A list of figures that have associated raw data
- A description of any restrictions on data availability

The raw and processed imaging datasets, as well as the supplementary data that support the findings of this study, are shared via a public repository in the Open Science Framework (https://osf.io/eq2ba/). The DOI of this dataset is 10.17605/OSF.IO/EQ2BA. The raw imaging datasets will also be shared via the OpenNeuro platform (https://openneuro.org/).

Field-specific reporting

Please select the one below that is the best fit for your research. If you are not sure, read the appropriate sections before making your selection.

Behavioural & social sciences study design

All studies must disclose on these points even when the disclosure is negative.

Study description	This is a quantitative experimental study. We delivered naturalistic auditory stimuli to healthy human participants during fMRI scans. The purpose of this study is to train an voxel-wise encoding model to predict fMRI responses during natural language comprehension and to analyze the cortical patterns of semantic processing of various word information.
Research sample	19 healthy human subjects (Purdue University students, all right-handed, all native English speakers,11 females, age 24.4±4.8) participated in this study. The rational of chosen study sample is to include all volunteers that meet the minimum requirements (native English speaker and normal hearing).
Sampling strategy	Sample size: 11 hours of naturalistic auditory story stimuli including 47,356 words (or 5,228 words if duplicates were excluded). This sample size is sufficient for fitting the predictive model in this study, since we used multiple linear regression analysis with 300 variables and around 28,000 observations (or time points).<br>No exact calculation was performed to predetermine the sample size since we used naturalistic stimuli. And the sample size per subject was determined based on the length of a continuous MRI scan session (around 2 hours).
Data collection	T1 and T2-weighted MRI and fMRI data were acquired in a 3T MRI system (Siemens, Magnetom Prisma, Germany) with a 64-channel receive-only phased-array head/neck coil. The auditory stimuli were presented through binaural MR-compatible headphones (Silent Scan Audio Systems, Avotec, Stuart, FL).<br>The authors Y.Z. and K.H. performed the data collection. They were not blinded to the experimental condition or the study hypothesis.
Timing	Data was collected from Feb 2018 to Jul 2018.
Data exclusions	No data was excluded from the analyses.
Non-participation	No participants declined participation.
Randomization	Participants were not allocated into experimental groups.

Reporting for specific materials, systems and methods

We require information from authors about some types of materials, experimental systems and methods used in many studies. Here, indicate whether each material, system or method listed is relevant to your study. If you are not sure if a list item applies to your research, read the appropriate section before selecting a response.

Materials & experimental systems

n/a	Involved in the study	
☒	☐	Antibodies
☒	☐	Eukaryotic cell lines
☒	☐	Palaeontology
☒	☐	Animals and other organisms
☐	☒	Human research participants
☒	☐	Clinical data

Methods

n/a	Involved in the study	
☒	☐	ChIP-seq
☒	☐	Flow cytometry
☐	☒	MRI-based neuroimaging

Human research participants

Policy information about studies involving human research participants

Population characteristics	See above.
Recruitment	The flyers (for recruiting volunteers) were attached to multiple buildings at Purdue University and were also announced via an online platform (Purdue Today) to all students and staffs at Purdue University. The participants were informed with the study purpose and MRI safety information before they got recruited. All participants were Purdue University students. This may introduce a bias to the results since they have high level of education.
Ethics oversight	All subjects provided informed written consent according to a research protocol approved by the Institutional Review Board at Purdue University.

Note that full information on the approval of the study protocol must also be provided in the manuscript.

Magnetic resonance imaging

Experimental design

Design type	Task-state; Naturalistic auditory stimuli.
Design specifications	A single story was presented in each fMRI session (6 mins 48 secs ± 1 min 58 secs). For each story, two repeated sessions were performed for the same subject. Each subject was scanned for 6 or 8 sessions.
Behavioral performance measures	The participants self-reported their level of understanding (scored 1-10) of the story after each session. All participants reported a score higher than 8. This behavioral measure was only used to check the experiment process and was not used in the data analyses.

Acquisition

Imaging type(s)	functional MRI
Field strength	3T
Sequence & imaging parameters	multiband = 8, 72 interleaved axial slices, isotropic 2mm spatial resolution, TR = 720 ms, TE = 31 ms, flip angle = 52°, field of view = 21 × 21 cm.
Area of acquisition	A whole brain scan was used.

Diffusion MRI ☐ Used ☒ Not used

Preprocessing

Preprocessing software	The MRI and fMRI data was preprocessed through HCP minimal preprocessing pipelines (version 3.4.0). The fMRI data were further spatially smoothed by using a gaussian surface smoothing kernel with a 2mm standard deviation.
Normalization	Non-linear transformation/volumn registration.
Normalization template	MNI152
Noise and artifact removal	The first 12 volumes and the last 3 volumes of each session was discarded. The voxel-wise fMRI signal was further detrended (4th order polynomial) and standardized (zero mean and unitary standard deviation) within each session and was averaged across repeated sessions.
Volume censoring	We did not do volume censoring.

Statistical modeling & inference

Model type and settings	We used a voxel-wise encoding model, which is a predictive model fitted by multiple linear regression with L2 regularization.
Effect(s) tested	We tested the effects of individual variance and stimuli variance with a 2-way ANOVA.

Specify type of analysis: ☐ Whole brain ☐ ROI-based ☒ Both

Anatomical location(s): Pre-defined ROIs in the human brainnetome atlas (Fan et al. 2016).

Statistic type for inference (See Eklund et al. 2016)	Voxel-wise analysis.
Correction	FDR was used for multiple comparison correction.

Models & analysis

n/a	Involved in the study
☐	☒ Functional and/or effective connectivity
☒	☐ Graph analysis
☐	☒ Multivariate modeling or predictive analysis

Functional and/or effective connectivity	Pearson correlation was used for measuring the connectivity and prediction accuracy.
Multivariate modeling and predictive analysis	We extracted the semantic features of the story stimuli from the pretrained word2vec model and represented each word as a 300-dimensional vector. We trained a linear model to use word features to predict fMRI response when subjects were listening to natural stories. We further applied this model to thousands of new words and mapped the cortical patterns for different semantic categories and semantic

relations through both voxel-wise univariate analysis and multivariate pattern analysis.
